# US Department of Defense–Funded Fate and Transport Research on Per‐ and Polyfluoroalkyl Substances at Aqueous Film–Forming Foam–Impacted Sites

**DOI:** 10.1002/etc.4694

**Published:** 2020-06-02

**Authors:** Richard H. Anderson, Timothy Thompson, Hans F. Stroo, Andrea Leeson

**Affiliations:** ^1^ Air Force Civil Engineer Center, San Antonio Texas USA; ^2^ SEE, Seattle Washington USA; ^3^ Stroo Consulting, Ashland Oregon USA; ^4^ Strategic Environmental Research and Development Program/Environmental Security Technology Certification Program Office Arlington Virginia USA

## INTRODUCTION

**Figure 1:**
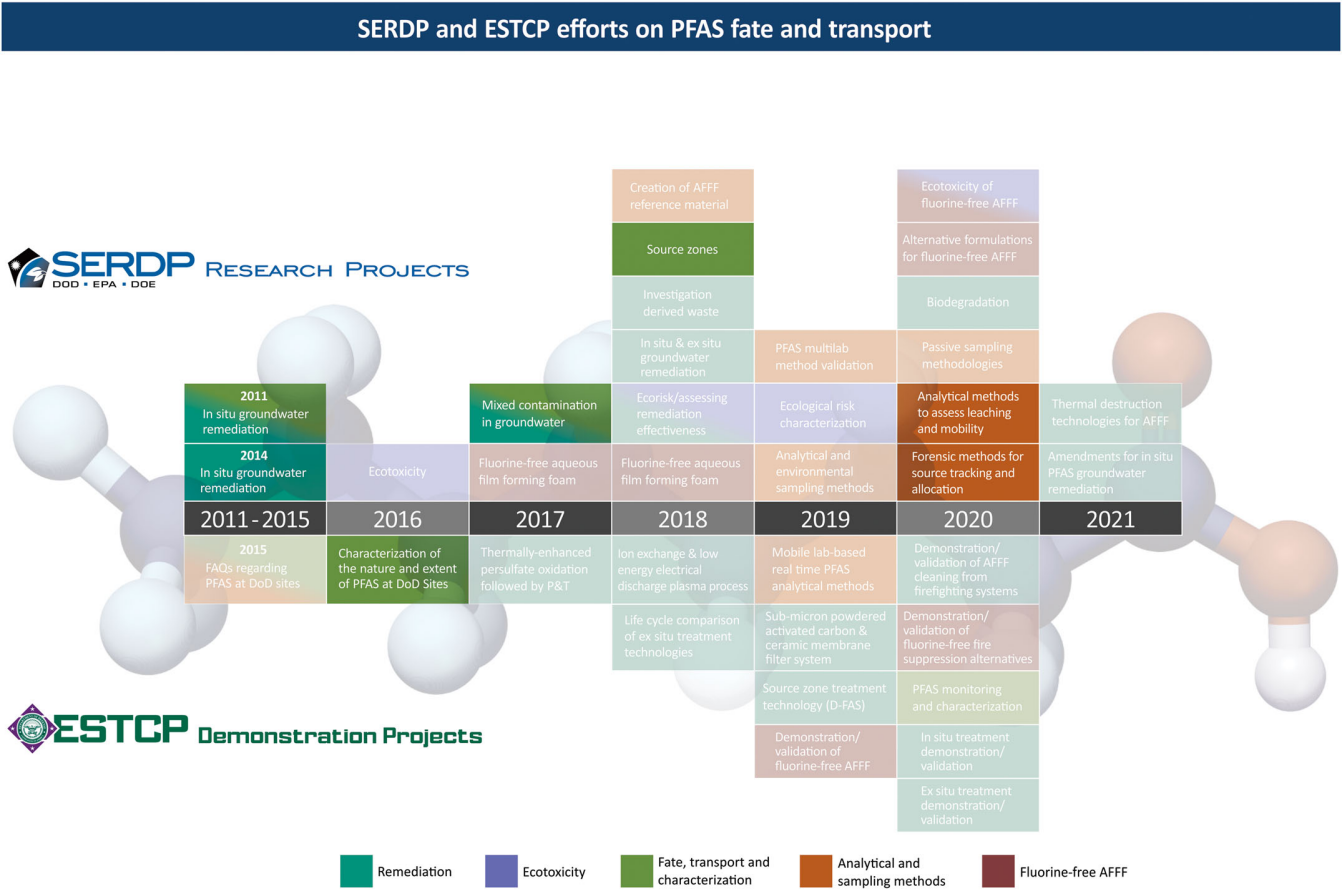
Chronology of Strategic Environmental Research and Development Program statements of need and Environmental Security Technology Certification Program projects related to the environmental fate and transport of per‐ and polyfluoroalkyl substances. See Table 1 for a description of each project. AFFF = aqueous film–forming foam; DoD = Department of Defense; ESTCP = Environmental Security Technology Certification Program; PFAS = per‐ and polyfluoroalkyl substances; P&T = Pump and Treat; SERDP = Strategic Environmental Research and Development Program.

The US Department of Defense (DoD) has made significant investment in understanding the fate and transport of per‐ and polyfluoroalkyl substances (PFAS) in the subsurface, primarily through the Strategic Environmental Research and Development Program (SERDP) and the Environmental Security Technology Certification Program (ESTCP). The SERDP is the DoD's environmental science and technology program and invests across a broad spectrum of basic and applied research, whereas ESTCP is the DoD's environmental technology demonstration and validation program and is intended to collect cost and performance data to overcome implementation barriers. Through these 2 programs, the DoD has funded a variety of projects to date related to the occurrence, transformation, and retention of PFAS in both the unsaturated and saturated subsurface, which have proven critical to the development of accurate conceptual site models (CSMs) and optimal remedial strategies. The chronology of SERDP/ESTCP projects related to PFAS fate and transport can be found online at https://www.serdp‐estcp.org/ (see “Featured Initiatives” tab located on the website), and is highlighted in Figure 1. This Focus article is intended to summarize this work.

**Figure 2:**
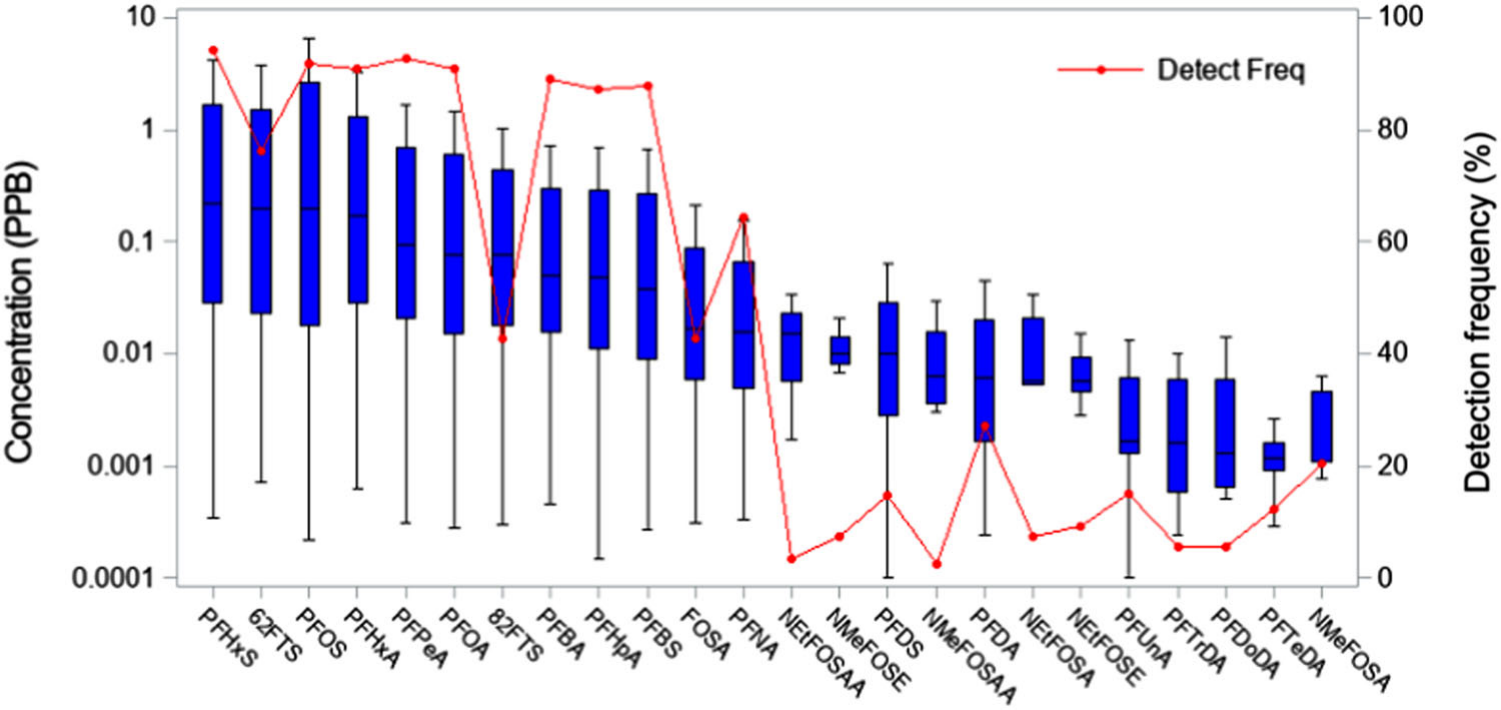
Concentrations and detection frequencies of routinely measured per‐ and polyfluoroalkyl substances in groundwater within source zones at US Air Force aqueous film–forming foam–impacted sites. FOSA = perfluorooctanesulfonamide; 62FTS = 6:2‐fluorotelomer sulfonate; 82FTS = 8:2‐fluorotelomer sulfonate; NEtFOSA = *N*‐ethylperfluorooctanesulfonamide; NEtFOSAA = *N*‐ethylperfluorooctanesulfonamidoacetic acid; NEtFOSE = *N*‐ethylperfluorooctanesulfonamidoethanol; NMeFOSA = *N*‐methylperfluorooctanesulfonamide; NMeFOSAA = *N*‐methylperfluorooctanesulfonamidoacetic acid; NMeFOSE = *N*‐methylperfluorooctanesulfonamidoethanol; PFBA = perfluorobutanoic acid; PFBS = perfluorobutanesulfonic acid; PFDA = perfluorodecanoic acid; PFDoDA = perfluorododecanoic acid; PFDS = perfluorodecanesulfonic acid; PFHpA = perfluoroheptanoic acid; PFHxA = perfluorohexanoic acid; PFHxS = perfluorohexanesulfonic acid; PFNA = perfluorononanoic acid; PFOA = perfluorooctanoic acid; PFOS = perfluorooctanesulfonic acid; PFPeA = perfluoropentanoic acid; PFTeDA = perfluorotetradecanoic acid; PFTrDA = perfluorotridecanoic acid; PFUnA = perfluoroundecanoic acid.

The need for PFAS fate and transport research has become more pressing in recent years as the DoD has recently committed to investigating the occurrence and environmental distribution of PFAS at thousands of potentially contaminated sites where aqueous film–forming foam (AFFF) was used. These AFFF‐impacted sites are characterized by a variety of operational scenarios involving AFFF since 1970 (see Interstate Technology Regulatory Council 2018 for additional detail). A current summary of groundwater concentrations for those PFAS routinely reported by DoD‐accredited commercial laboratories observed within representative source zones is provided in Figure 2; perfluorohexane sulfonic acid and perfluorooctane sulfonic acid are among those PFAS observed with the greatest concentrations and detection frequencies (>90%), suggesting that electrochemical fluorination–based AFFF was historically used at most sites (Interstate Technology Regulatory Council 2018 provides additional detail). Although the DoD is currently focused primarily on confirming PFAS contamination at all AFFF‐impacted sites and mitigating any resulting potential for human exposure, program‐wide efforts to delineate the full extent of all PFAS contamination, to include source strength evaluations and human and ecological risk assessments, are forthcoming.

The SERDP/ESTCP‐funded research on PFAS fate and transport began in 2011 (Figure 1) when it became obvious that AFFF formulations were complex and that little progress could be made toward site remediation without understanding their composition and behavior in the subsurface. Although most of these projects are still in progress, the earliest projects have been completed. Early pioneering projects were led by Jennifer Field from Oregon State University and Chris Higgins from the Colorado School of Mines and have generated a foundation of knowledge for the research that has followed. Recent projects have been guided by a 2017 workshop on environmental PFAS research needs (Strategic Environmental Research and Development Program 2017), the results of which are summarized herein as they relate to fate and transport (Textbox 1). The following review of SERDP/ESTCP‐funded research in this area is not intended as a thorough summary of the entire literature but rather provides insight into the unique problems posed by AFFF‐impacted sites and the current status of the DoD efforts to solve those problems.

TextBox 1 **Summary of the 2017 Strategic Environmental Research and Development Program/Environmental Security Technology Certification Program workshop related to the environmental fate and transport of per‐ and polyfluoroalkyl substances**

**Table:** 

Data gap	Summary
Process understanding	A scientifically sound approach to performing site characterization was, and remains, a pressing need given the pace of the Department of Defense's response efforts and the current uncertainties. In particular, reliable predictions of the source strength within vadose zones, aquifers, and sediments are not currently possible. As such, mass discharge from these sources over time is difficult to measure and model.
Phase partitioning	Partitioning of per‐ and polyfluoroalkyl substances (PFAS) to solids, water, and air may affect remedial technology selection and efficacy; and, conversely, remediation methods may affect PFAS partitioning. There is a lack of information on the underlying partitioning processes for many PFAS, and unlike many traditional groundwater contaminants, PFAS are surface‐active substances and, therefore, tend to accumulate at air–water, water–solvent, and soil–water interfaces, greatly complicating characterization efforts.
Predictive models	Models would be helpful for evaluating the potential migration of perfluoroalkyl acids and the concentrations and composition of the PFAS mixtures over time. These models should incorporate precursor biotransformations and the effects of environmental conditions such as redox, pH, salinity, organic carbon content, interfacial effects, and cocontaminant effects on fate and transport. Developing and validating such models will improve conceptual site models and decisions regarding the need for further investigations and/or remediation.
Leachability methods	Given the difficulties in predicting transport, methods to measure potential PFAS migration directly in unsaturated or saturated soils are needed. It is particularly important to assess the potential for migration to groundwater from residual contamination located in the vadose zone. Determining whether residual sources pose a continuing risk after the most mobile constituents have been depleted requires validated leachability methods for use in soils and sediments.

## KEY FATE AND TRANSPORT QUESTIONS

Fundamental fate and transport questions critical for AFFF‐impacted site management that have guided SERDP/ESTCP investments include the following. 1) What is the composition of AFFF formulations? Understanding AFFF composition (both the PFAS and non‐PFAS constituents) is important to focus research on the most important perfluoroalkyl acids (PFAAs) present at AFFF‐impacted sites, as well as other PFAS and non‐PFAS constituents that are or may eventually be of regulatory concern. 2) Can we measure and predict the magnitude of PFAS fate and transport processes? The cumulative (and potentially interactive) effects of all applicable processes may mitigate or in some cases exacerbate transport. Accurate models, even at the screening level, would help managers faced with prioritizing sites and designing remediation strategies. 3) What is the fate of PFAA precursors in various AFFF formulations under different conditions? Precursors can represent a significant proportion of the total PFAS mass in some AFFFs and may represent an ongoing environmental source of PFAAs. 4) How do non‐PFAS AFFF constituents affect PFAS fate and transport? Non‐PFAS AFFF constituents can potentially sequester PFAS, may facilitate transport as a cosolvent, or may compete for sorption/retention sites. If biodegradable, they also may consume electron acceptors, preventing or delaying precursor biotransformation. 5) What measurements and monitoring methods should the DoD use to adequately characterize AFFF‐impacted sites? Given the DoD's aggressive plans for investigating thousands of sites and the current lack of accurate models, there is an urgent need for characterization guidance to foster efficient delineation efforts and inform management decisions.

## FUNDED PROJECTS

The first SERDP statement of need (SON) relevant to PFAS fate and transport following the 2017 workshop focused on AFFF source zones. The overall goal was to improve predictions and measurements of vadose zone processes over time. Six projects were funded under this SON, each comprising different strategies to interrogate the various processes and develop predictive models useful for different purposes. General objectives were to investigate 1) the fate of precursor PFAS, and 2) the extent to which residual PFAAs retained throughout vadose zone soils contribute to groundwater contamination. These projects are briefly summarized individually in Table 1. All 6 projects are ongoing.

**Table 1:** Summary of Strategic Environmental Research and Development Program–funded projects related to per‐ and polyfluoroalkyl substance fate and transport

Project title	Primary objectives	Project^a^
Behavior of Perfluoroalkyl Chemicals in Contaminated Groundwater^b^	The overall goal of this pioneering research was to evaluate the relative importance of key physicochemical and biological parameters in determining the fate and transport of PFAAs in groundwater in the presence of cocontaminants and during remediation of cocontaminants.	ER‐2126
Characterization of the Fate and Biotransformation of Fluorochemicals in AFFF‐Contaminated Groundwater at Fire/Crash Testing Military Sites^b^	The overall goals of this pioneering research were to identify individual PFAS and their oxidizable precursors in AFFF formulations and AFFF‐contaminated groundwater, sediment, and soil and to carry out biotransformation studies to determine precursor biodegradation pathways in AFFF‐contaminated media.	ER‐2128
Key Fate and Transport Processes Impacting the Mass Discharge, Attenuation, and Treatment of Poly‐ and Perfluoroalkyl Substances and Comingled Chlorinated Solvents or Aromatic Hydrocarbon^c^	Specific objectives of this project include 1) investigation of the fundamental mechanisms controlling the release of PFAS from complex source zone phases, 2) examination of the coupled diffusion and potential abiotic reactions of PFAS and comingled contaminants in low‐permeability materials, 3) assessment of the biotic transformation of the wide range of PFAS and cocontaminants (chlorinated solvents and BTEX) present in the dissolved plume and the impacts of PFAS on cocontaminant bioremediation, and 4) quantification of the impacts of remedial activities targeting cocontaminants (i.e., BTEX, chlorinated solvents) on the PFAS plume.	ER‐2720
Insights into the Long‐Term Mass Discharge & Transformation of AFFF in the Unsaturated Zone^c^	Field and laboratory data are being used to elucidate processes affecting PFAS transformation and mass discharge of AFFF from vadose zone sources over time. Efforts employ the use of a highly characterized and instrumented field test cell emplaced within an aged unsaturated zone AFFF source zone. The test cell is used to facilitate enhanced water flushing of the unsaturated zone source, thereby serving as a means to evaluate PFAS discharge, composition, and transformation as a function of time and fractional PFAS removal. Parallel bench‐scale testing is being performed to provide additional mechanistic insight needed to interpret the field results—specifically, to assess soil desorption kinetics, PFAS partitioning to natural colloids, micellar PFAS mass discharge, and PFAA precursor transformation under cyclic aerobic–anaerobic conditions.	ER18‐1204
Establishment of Fate and Transport Mechanics for PFAS under Controlled Aquifer Conditions and Correlation to Existing Data^c^	The technical objective of this project is to utilize a scaled aquifer, where chemistry and aquifer conditions can be controlled, to determine relevant factors controlling the transport of PFAS in the subsurface.	ER18‐1621
Development and Laboratory Validation of Mathematical Modeling Tools for Prediction of PFAS Transformation, Transport, and Retention in AFFF Source Areas^c^	This project integrates experimental and modeling data designed to 1) improve understanding of sequestration mechanisms and abiotic/biotic transformations controlling transport and persistence of selected PFAS and 2) develop and validate mathematical models on the processes governing transformation, transport, and retention of PFAS in AFFF source areas. Laboratory experiments on the transport and partitioning experiments and coupled abiotic and biotic transformations will provide data necessary to develop and validate mathematical modeling tools.	ER18‐1149
A Mechanistic Understanding of PFAS in Source Zones: Characterization and Control^c^	The study is designed to identify fundamental mechanisms controlling the nature and permanence of PFAS interactions with components of source zone soils/sediments under unsaturated and saturated conditions and in the presence of NAPL. Controlled laboratory column experiments will characterize the transport of anionic, cationic, and zwitterionic PFAS and their interactions with organic matter and mineral phases of pristine soils/sediments and with PFAS‐coated soils/sediments. Additional experiments will be done to characterize the number and type of thermodynamically stable phases that form when AFFFs are mixed with common NAPLs, such as JP4 and trichloroethene, and their impact on PFAS transport under saturated conditions. These data will be used then to assess PFAS mobility under unsaturated conditions in the vadose zone to identify the key hydraulic parameters that control PFAS mobility and retention.	ER18‐1259
Evaluating the Importance of Precursor Transport and Transformation for Groundwater Contamination with PFAS^c^	Mobility of PFAS and PFAA precursors in groundwater is being evaluated through a series of controlled column experiments. Partition coefficients for PFAS, with a focus on geochemical conditions that may affect mobility of PFAS, will be determined. Biotransformation of PFAA precursors will be examined to determine rates and natural factors that can affect conditions. Quantifying these conditions and reaction rates will contribute to the development of a reactive transport model. Passive sampling methods are also being developed that will provide time‐weighted average concentrations of PFAS traveling from the source unsaturated zone to the groundwater and monthly average PFAS concentrations and correlations with seasonal and associated geochemical variations in surface waters.	ER18‐1280
Baseline Data Acquisition and Numerical Modeling to Evaluate the Fate and Transport of Per‐ and Polyfluoroalkyl Substances within the Vadose Zone^c^	Specific objectives of this proof‐of‐concept project include 1) to generate fundamental physicochemical properties data with which to evaluate the significance of air–water interfacial accumulation as a source of retention and as an environmental sink for PFAS, 2) to investigate the potential for interfacial tension‐induced flow and lateral spreading of PFAS in the vadose zone to determine whether these processes could increase the scale of PFAS contamination, and 3) to incorporate these mechanisms into the current version of the commercially available HYDRUS unsaturated flow and transport model.	ER18‐1389
Development and Validation of Novel Techniques to Assess Leaching and Mobility of Per and Polyfluoroalkyl Substances (PFAS) in Impacted Media^d^	The overarching goal of this project is to develop a framework for evaluation and prediction of the release of PFAS from AFFF‐impacted media. The specific objectives include 1) development of a standard leaching assessment methodology for AFFF‐impacted media; 2) utilization of approaches including HRMS, mid‐infrared spectroscopy, and chemometrics to evaluate and develop a predictive model of PFAS sorption and desorption to AFFF‐impacted media; and 3) comparison of results of laboratory testing to leaching and mobility under field‐relevant conditions to develop an approach for translation of bench‐scale test results to site‐scale implications.	ER20‐1126
Improving Access and Utility of Analytical Data for the Confident Discovery, Identification, and Source‐Attribution of PFAS in Environmental Matrices^d^	The objective of the proposed research is to develop a data analytics infrastructure to contain PFAS mass spectral information and metadata of detected PFAS. With an open database structure, analytical laboratories will be able to include this database in their nontargeted analytical analysis workflow to identify unknown PFAS.	ER20‐1056
Establishing an Approach to PFAS Forensics and a PFAS Source Materials Forensic Library^d^	The overall objective of this work is to provide a framework for PFAS forensics in environmental samples. The 3 steps to achieving this objective include 1) establishing a PFAS forensic library of source materials; 2) validating PFAS forensic approaches using environmental samples and determining the extent to which forensic information is preserved in environmental samples, and 3) characterizing changes to select PFAS forensic signatures associated with environmental fate and transport.	ER20‐1121
Machine Learning Pattern Recognition for Forensic Analysis of Detected Per‐ and Polyfluoroalkyl Substances in Environmental Samples^d^	This 1‐yr project will explore the use of modern machine learning algorithms to evaluate the probability that PFAS in environmental samples come from AFFF sources. The approach will use machine learning to search for recognizable patterns in PFAS‐containing samples, with the objective of assigning probabilities that the contamination originates from specific sources.	ER20‐1205
Ultrahigh‐Resolution Fourier‐Transform Ion Cyclotron Resonance Mass Spectrometry for Fingerprinting, Source Tracking, and Allocation of Per‐ and Polyfluoroalkyl Substances (PFAS)^d^	The overarching objective of this 1‐yr project is to apply ultrahigh‐resolution Fourier transform ion cyclotron resonance mass spectrometry to identify compounds in AFFFs at the molecular level that can be used to guide the development of novel analytical approaches to identify unique marker compounds for AFFF “fingerprinting” and PFAS source allocation and to catalogue PFAS associated with AFFF releases.	ER20‐1265
A Simple and Robust Forensic Technique for Differentiating PFAS Associated with AFFF from other PFAS Sources^d^	This project consists of the following 3 specific objectives: 1) expand the total oxidizable precursor assay to obtain more detailed information on polyfluorinated compounds present at contaminated sites to support a proposed forensic tool, 2) identify data that are most useful to the analysis of PFAS source fingerprints, and 3) assess the effect of environmental processes (e.g., biotransformation, sorption) on observed fingerprints at AFFF‐impacted sites.	ER20‐1330
Comprehensive Forensic Approach for Source Allocation of Poly‐ and Perfluoroalkyl Substances^d^	This project will develop a set of tools for use in forensic PFAS source allocation, including 1) a database of PFAS and *other chemical constituents* observed in discrete PFAS sources (i.e., landfill leachate, municipal wastewater effluent, chromium plating, etc.), 2) a comprehensive PFAS transformation pathway map to establish the context and linkages between specific PFAS and discrete sources, 3) a multivariate analysis resulting in an LC‐MS/MS‐based “forensic LC‐MS/MS PFAS panel” for use in PFAS source allocation, and 4) a curated HRMS PFAS library to enable more precise source allocations when needed.	ER20‐1375

^a^ Web links to project summaries are provided as available.

^b^ Completed projects.

^c^ Ongoing projects.

^d^ New‐start projects.

AFFF = aqueous film–forming foam; BTEX = benzene, toluene, ethylbenzene, and xylene; HRMS = high‐resolution mass spectrometry; NAPL = nonaqueous phase liquids; PFAA = perfluoroalkyl acid; PFAS = per‐ and polyfluoroalkyl substances.

Two subsequent SERDP SONs have resulted in funded projects specific to fate and transport (Figure 1). The first SON aimed to develop improved forensic methods and tools for PFAS source tracking and allocation. General objectives included 1) evaluation of conventional or novel analytical techniques or methodologies to differentiate PFAS from AFFF versus non‐AFFF sources; 2) development of spectral libraries of PFAS to include both AFFF‐derived PFAS as well as PFAS derived from other sources; and 3) development of improved analytical methods and/or validated models to predict changes to AFFF mixtures over time, including chemical pathways to the most toxic compounds. Six projects were funded under this SON as well and are individually summarized in Table 1. All 6 projects will be initiated in 2020.

The other recent applicable SON is specific to analytical methods to assess the leaching and mobility of PFAS. Standard operating protocols for such methods would be helpful for screening soils and sediments for treatment and/or disposal. The goal was to develop and validate a standard analytical method, similar to the Synthetic Precipitation Leaching Procedure (US Environmental Protection Agency 1994) commonly used for other contaminants, that could be used to support decision‐making for PFAS site investigations and source zone management and possibly to evaluate stabilization and soil‐washing treatment technologies. One project (ER20‐1126) was selected under this SON and will be initiated in 2020 (see Table 1 for more detail).

Only 2 ESTCP projects specific to PFAS fate and transport have been funded to date. Project ER20‐1633 is ongoing and intended to provide a case study of soil and groundwater delineation at an AFFF‐impacted site, with a careful accounting of mass distribution between the vadose and saturated zones, including PFAAs and precursors. Site‐specific factors (e.g., organic carbon content, ion exchange capacity, redox conditions) that mitigate PFAS transport will be demonstrated at field scale utilizing an array of novel characterization tools, including the first demonstration of particle‐induced gamma‐ray emission spectroscopy to measure total organic fluorine in environmental matrices and the total oxidizable precursor assay. The former fire‐training area under investigation also is located near a former wastewater‐treatment facility, which may enable fingerprinting of AFFF‐based sources compared to non‐AFFF sources. The results are intended to provide a protocol for efficient AFFF site investigations and quantitative CSMs to guide selection of mitigation and remediation strategies. Finally, the detailed plume characterization will provide information on transformation pathways and estimates of natural attenuation under prevailing groundwater redox conditions that can be generalized across the DoD's portfolio of AFFF‐impacted sites.

The other ESTCP project (ER20‐5088) is a new start and aims to attain insight into the transformation and mass discharge of PFAS from AFFF sources that reside in the vadose zone and capillary fringe and to understand how these processes change over time with AFFF composition and mass. Lysimetry will be used to sample soil water to focus on the unique processes that occur in the vadose zone (e.g., varying saturation as a result of wetting and drying, aerobic/anaerobic cycling, creation of air–water interfaces, and release of natural colloids) at field sites. A key aspect of this research is to assess, describe, and quantify the relationship between PFAS mass removal and mass discharge (i.e., the source strength). Determining the processes that control the source strength and identifying methods to characterize this relationship for AFFF‐impacted sites would greatly improve site characterization approaches.

## SUMMARY

The research to date demonstrates that AFFF‐impacted sites have unique characteristics that complicate PFAS fate and transport. Different AFFF formulations were used over time, and all of these contained many different PFAS, as well as a wide range of other constituents that can interact with these PFAS. There is still uncertainty regarding the full composition and sources of PFAS and cocontaminants among the portfolio of DoD sites, but there is consensus specifically regarding the following: 1) historic AFFF discharges exclusively occurred at the ground surface given the operational context, 2) retention of PFAS in soil is significant (Anderson et al. 2019), and 3) aerobic biological pathways dominate the transformation of precursors in soil and the subsurface (see Interstate Technology Regulatory Council 2018 for a summary of relevant literature). Therefore, vadose zone processes are critical to accurate CSMs and, consequently, have become a priority topic area of the PFAS fate and transport research funded by SERDP/ESTCP.

Although much of the SERDP/ESTCP‐funded research on PFAS retention is ongoing, the work to date (and other published research) has resulted in several overarching conclusions. Specifically, soil retention of PFAS is primarily affected by 1) the specific chemical and physical properties of the individual PFAS to specifically include the perfluorinated chain length and functional group (e.g., Anderson et al. 2016); 2) pH‐dependent electrostatic sorption to various clay minerals (e.g., Li et al. 2019); 3) hydrophobic sorption to soil organic matter (e.g., Anderson et al. 2019), specifically including the proteinaceous components (Li et al. 2019); and 4) fluid–fluid interactions, including both air–water and nonaqueous phase liquid–water interfacial sorption (e.g., Costanza et al. 2019; Schaefer et al. 2019). Moreover, these processes are all enhanced to some extent in soil solution relative to reagent water (typically utilized in experimental studies for simplicity) because of pH, ionic strength, and cation composition effects (e.g., Costanza et al. 2019; Schaefer et al. 2019). Collectively, these conclusions demonstrate the novelty and criticality of PFAS fate and transport processes to accurate CSMs and remedial strategies.

Complicating factors to field‐scale predictions, however, currently are thought to include 1) lack of methods to assess the rate and extent of precursor biotransformation; 2) nonideal PFAA transport behavior attributable to complex sorption/desorption kinetics, resulting in attenuation over time (e.g., Brusseau et al. 2019); and 3) greater retardation of linear versus branched isomers of a given PFAS (e.g., Kärrman et al. 2011). Additional uncertainty highlighted in recent literature results from the extrapolation of experimental observations (typically performed at relatively high concentrations because of experimental constraints) down to environmentally relevant concentrations that are highly dependent on the selected sorption isotherm (Schaefer et al. 2019). Moreover, the various retention processes have to date been studied independently but could potentially interact in some way depending on transient site conditions (e.g., soil moisture content). Clearly, research funded by SERDP/ESTCP and others has overwhelmingly demonstrated the complexity of AFFF‐derived PFAS behavior in soil and the subsurface and, thus, the critical need for additional studies, in particular studies that provide rigorous field validation of transport models in support of quantitative groundwater mass discharge estimates.

## Data Availability

Data are available from Richard Anderson (Richard.anderson.55@us.af.mil).
